# Development of VRE meningitis due to haematogenous spread while on high-dose daptomycin therapy

**DOI:** 10.1093/jacamr/dlad118

**Published:** 2023-11-17

**Authors:** Srivani Sanikommu, Katherine Lusardi, Lyle Burdine, Ryan K Dare

**Affiliations:** Department of Internal Medicine, Division of Infectious Diseases, University of Arkansas for Medical Sciences, 4301 W Markham Slot #639, Little Rock, AR, USA72205-7101; Baptist Health Medical Center, Little Rock, AR, USA; Department of Surgery, University of Arkansas for Medical Sciences, 4301 W Markham Slot #639, Little Rock, AR, USA72205-7101; Department of Internal Medicine, Division of Infectious Diseases, University of Arkansas for Medical Sciences, 4301 W Markham Slot #639, Little Rock, AR, USA72205-7101

## Abstract

**Introduction:**

Vancomycin-resistant *Enterococcus faecium* (VRE) meningitis accounts for only 0.3%–4.0% of bacterial meningitis cases and typically occurs following neurosurgical intervention. We describe a rare case of a patient without neurological devices *in situ* or a recent neurological procedure who developed VRE meningitis via haematogenous spread. Optimal treatment for VRE meningitis is unknown.

**Case presentation:**

A 67-year-old male with end-stage liver failure underwent liver transplantation complicated by VRE bacteraemia and subsequently developed VRE meningitis while on high-dose daptomycin therapy (12 mg/kg/day). Due to clinical and microbiological failure with daptomycin, he was switched to linezolid and symptoms resolved rapidly. He completed 2 weeks of linezolid, fully recovered, and continued to do well without complications at the 5 month follow-up.

**Conclusions:**

This case highlights the severity of VRE infections in solid organ transplant recipients and raises concerns about daptomycin penetration into the CNS. Linezolid could be considered the preferred treatment for VRE CNS infections rather than high-dose daptomycin.

## Case description

A 67-year-old man with end-stage liver failure due to primary sclerosing cholangitis underwent liver transplantation without immediate postoperative complications. Seven days after the transplant, he developed a fever (38.5°C) and hypotension, requiring transfer to the ICU for management of septic shock. He was intubated for airway protection, started on continuous renal replacement therapy due to acute renal failure (serum creatinine 4.1 mg/dL, baseline 1.1 mg/dL), and was initiated on vancomycin, meropenem and micafungin. Abdominal CT revealed a 9.7 × 6.2 cm fluid collection in the hepatorenal recess concerning the abscess. Vancomycin-resistant *Enterococcus faecium* (VRE: daptomycin MIC 2 mg/L; linezolid MIC 1 mg/L) was isolated from blood culture, so vancomycin was switched to daptomycin (8 mg/kg/day), which was preferred due to the risk of cytopenias with linezolid. Repeat blood cultures after 48 h again grew VRE (daptomycin MIC 4 mg/L), and the daptomycin dose was increased to 12 mg/kg/day. His blood cultures cleared on high-dose daptomycin, and a trans-oesophageal echocardiogram revealed no valvular vegetation. Notably, aspiration from the hepatorenal recess collection obtained 8 days after the onset of sepsis grew VRE (daptomycin MIC 4 mg/L), for which a drain was placed. The patient had no signs of CNS involvement until 9 days after the onset of sepsis and 4 days after clearance of blood cultures, when he developed altered mentation with seizure-like activity, nystagmus and persistent high-grade fevers (40°C). This patient had no known structural CNS abnormalities and, other than being immunosuppressed, had no known underlying physiological reasons to develop meningitis. Brain MRI showed no acute intracranial process although CSF analysis revealed a pleocytosis with 2051 leucocytes/μL (90% neutrophils), elevated protein (161 mg/dL; normal range 15–45 mg/dL), and low glucose 39 mg/dL as serum glucose was 150 mg/dL (normal range 40–70 mg/dL). Molecular testing of CSF for herpes simplex 1 and 2, varicella zoster virus, cytomegalovirus, enterovirus, parechovirus, human herpes virus 6, *Cryptococcus*, *Streptococcus agalactiae*, *Streptococcus pneumoniae*, *Listeria monocytogenes*, *Haemophilus influenzae*, *Neisseria meningitidis* and *Escherichia coli* were negative. CSF culture grew VRE (daptomycin MIC 4 mg/L; linezolid MIC 1 mg/L). He was switched from daptomycin to linezolid 600 mg twice daily with rapid and significant clinical improvement. He completed 2 weeks of linezolid, made a full recovery, and was discharged home and continued to do well without complications at the time of 5 month follow-up.

## Discussion

VRE meningitis accounts for only 0.3%–4.0% of bacterial meningitis cases and is typically reported in patients with neurosurgical intervention and neurological device placement.^1–5^ Notably, these infections are associated with other enterococcal infections and colonization sites. Patients at high risk include immunocompromised individuals and hospitalized patients with chronic medical illnesses.^6,7^ Cases of VRE CNS infections reported in the literature are predominantly noted to be associated with indwelling neurological devices such as shunts, drains, extraventricular drains and those who underwent neurosurgical procedures, including subdural haematoma drainage, craniotomy, aneurysm clipping or tumour resection.^8–13^ Antibiotic treatment used for these cases has included chloramphenicol, quinupristin-dalfopristin, teicoplanin, rifampin, streptomycin, amikacin and linezolid with variable success.^14^ There are few cases of VRE meningitis due to haematogenous spread reported in the literature, highlighting the importance of this case presentation.

At our institution, approximately only 20% of hospital-acquired VRE isolates are susceptible to vancomycin, although >90% are susceptible to linezolid and susceptible dose dependent to daptomycin (R. K. Dare and S. Sanikommu, unpublished data). Antimicrobial susceptibility testing of blood, abscess fluid and CSF isolates obtained from this patient was susceptible to linezolid and susceptible dose dependent to daptomycin. High-dose daptomycin (≥8 mg/kg) is recommended for VRE infections to achieve adequate kill ratios.^15,16^ In our patient, using high-dose daptomycin (12 mg/kg/day) for VRE bacteraemia did not prevent haematogenous spread to CNS despite clearing the bacteraemia. Additionally, if the CNS had been seeded during initial bacteraemia and prior to initiating daptomycin, high-dose treatment did not prevent disease progression, resulting in microbiological and clinical failure. This raises concerns about daptomycin CSF penetration, where one study reported a range of only 9%–11%,^17^ and according to Piva *et al.,*^18^ daptomycin (up to 7 days at 10 mg/kg) has minimal penetration into CSF. Following the initiation of linezolid, this patient experienced rapid improvement in CNS symptomatology with the resolution of fevers and continued treatment for 14 days. Studies have shown that linezolid reaches mean CSF concentrations of 66% and 77%, suggesting good CSF penetration in critically ill neurosurgical patients.^19^

There have been reports of daptomycin resistance to VRE that developed while on daptomycin therapy.^20^ The patient described herein had progression of symptoms with persistent bacteraemia with an increase in daptomycin MIC from 2 mg/L to 4 mg/L on repeat blood cultures after 48 h of daptomycin therapy. Although a 1-fold dilution does not suggest the presence of inducible resistance, this finding might be clinically significant and perhaps an early sign of emerging daptomycin tolerance. According to Munita *et al.*,^21^ daptomycin MICs between 3 and 4 mg/L might harbour mutations in **LiaFSR** and could potentially lead to the development of daptomycin resistance. Further studies are needed to evaluate VRE resistance mechanisms in isolates with higher daptomycin MICs of 3–4 mg/L.

### Conclusion

We report a rare case of VRE meningitis due to haematogenous spread progressing despite treatment with high-dose daptomycin for 10 days. Symptoms resolved rapidly with initiation of linezolid therapy. This case highlights the severity of VRE infections in solid organ transplant recipients, and thus linezolid could be considered as preferred treatment for VRE CNS infections.
